# Multimodal decoding of error processing in a virtual reality flight simulation

**DOI:** 10.1038/s41598-024-59278-y

**Published:** 2024-04-22

**Authors:** Michael Wimmer, Nicole Weidinger, Eduardo Veas, Gernot R. Müller-Putz

**Affiliations:** 1 Know-Center GmbH, Graz, Austria; 2 Institute of Interactive Systems and Data Science, Graz University of Technology, Graz, Austria; 3 Institute of Neural Engineering, Graz University of Technology, Graz, Austria; 4 BioTechMed-Graz, Graz, Austria

**Keywords:** Biomedical engineering, Computer science

## Abstract

Technological advances in head-mounted displays (HMDs) facilitate the acquisition of physiological data of the user, such as gaze, pupil size, or heart rate. Still, interactions with such systems can be prone to errors, including unintended behavior or unexpected changes in the presented virtual environments. In this study, we investigated if multimodal physiological data can be used to decode error processing, which has been studied, to date, with brain signals only. We examined the feasibility of decoding errors solely with pupil size data and proposed a hybrid decoding approach combining electroencephalographic (EEG) and pupillometric signals. Moreover, we analyzed if hybrid approaches can improve existing EEG-based classification approaches and focused on setups that offer increased usability for practical applications, such as the presented game-like virtual reality flight simulation. Our results indicate that classifiers trained with pupil size data can decode errors above chance. Moreover, hybrid approaches yielded improved performance compared to EEG-based decoders in setups with a reduced number of channels, which is crucial for many out-of-the-lab scenarios. These findings contribute to the development of hybrid brain-computer interfaces, particularly in combination with wearable devices, which allow for easy acquisition of additional physiological data.

## Introduction

Interactions with a virtual environment (VE) can give rise to errors stemming from intentional design choices made by the simulation creators or unintentional factors. These errors encompass both intended and unintended aspects and may manifest as unexpected changes or behaviors within the VE, often contrary to the user’s intentions. An increasing number of head-mounted displays (HMDs) used to visualize such VEs have integrated cameras and sensors to measure physiological signals, like eye-tracking, pupil size, or heart rate (HR). In the present study, we investigate the potential impact of such additional physiological signals on the decoding of human error processing, which has been previously performed almost exclusively using electroencephalographic (EEG) data only^1^.

Since the early 1990s, research on error processing in the brain has developed from analyzing error-related potentials (ErrPs) after discrete mistakes in speed response tasks^2,3^ to studying erroneous actions in real-life environments, e.g., while navigating physical^4^ or virtual objects^5–8^. ErrPs are often characterized by two components appearing over frontocentral and centroparietal areas of the cortex, i.e., the error-related negativity (ERN), followed by an error positivity (Pe), shortly after error occurrence^9^. Additionally, multiple works reported an N400 component elicited by errors in human–computer interactions (HCIs)^6,10–13^. Source estimations suggest that these components are generated in the anterior cingulate cortex^6^, which is commonly found to be involved in error processing. Readers can refer to comprehensive reviews on the neural origin of these signals^14,15^.

Although most recent experiments exploring error processing use 2D paradigms^16–18^, emerging immersive technologies lead to increasing interest in studying ErrPs in virtual reality (VR). This interest is to some extent driven by potential flaws in HCIs, e.g., visualization errors, errors in the interaction with the interface, or misinterpretations of the user’s intentions. These flaws can impair the user experience. Possible strategies to address such problems include approaches from brain-computer interfaces (BCIs)^19^, where algorithms decoding ErrPs are implemented to allow the system to stop unintended actions or correct its behavior^20^. For such systems to be reactive, errors need to be detected online, i.e., in real-time, which has been demonstrated successfully^4,21^. Interested readers are referred to Chavarriaga et al.^22^ for an overview of ErrPs in BCIs. Additional strategies for corrective systems in immersive VR are dynamic adaptations of the visualizations and interactions, or the provision of supplemental visual aids to support the users^23^.

Subsequently, recent works investigate ErrPs in VR using HMDs for visualization. Errors in the interaction with the VR elicited ErrPs after participants lost control of objects in a tracking task, as well as giving erroneous feedback after successful task completion^24^. Similarly, Singh et al.^25^ and Gehrke et al.^26^ studied the modulation of ErrPs in the interaction with virtual objects. In these works, visual or haptic feedback was given after participants touched a visual cube. Premature feedback mimicking glitches in the VR led to ErrPs which could be decoded offline with an accuracy of 77% on a single-trial basis.

Further erroneous interactions with systems have been studied, e.g., suddenly displaced targets in aiming tasks or errors during the continuous control of virtual agents. The first was described as early as 2005 by Dietrichsen et al.^27^ in a joystick aiming task. Such target errors cause sudden discrepancies between actual and required motor commands following suddenly changing environments^28^. The second mimics misinterpretations of the users’ intentions by the system, as mentioned before. If participants cannot correct errors, outcome errors occur^7,29^. As these errors are part of numerous interactions and hence of general interest, respective brain responses have been studied extensively^22^.

In addition to brain responses, error processing has been shown to cause further physiological reactions. Previous works reported pupil dilation after the perception of erroneous events^30–32^, and could even find variations in the pupillometric responses for different types of error^33^. However, these works did not decode errors from pupil size signals. Several studies suggested that error processing also modulates cardiac activity. In response tasks, the HR was found to decelerate after erroneous decisions^32,34,35^. Even though there is extensive literature on physiological correlates of errors obtained in non-immersive scenarios, we identified a lack of attempts to utilize multimodal information^36^, e.g., both EEG and pupil size, to improve existing EEG-based systems for error detection.

A major limiting factor for the usability of such systems in practical applications is the preparation time needed to mount the EEG electrodes. One possible way to address this problem is to reduce the number of EEG electrodes to a minimum. Recently, Ancau et al.^37^ used a consumer-grade EEG headset to decode ErrPs from one channel. As reducing the number of channels usually leads to a performance decline, such systems might particularly profit from additional pupillometric information. Since we are not aware of previous research on hybrid error decoding incorporating pupillometric data, we could only speculate on the performance of such classifiers before the experiments. However, consistent reports of error-related pupil dilations indicate the possibility of performance improvements compared to error decoding using reduced EEG setups.

Consequently, this work aims to study the following research questions (RQs):

- **(RQ1)** Can we find physiological responses, i.e., brain, pupillometric, or cardiac responses, to target and interaction errors in an immersive VR scenario?
- **(RQ2)** Can pupil size data be used to decode error processing?
- **(RQ3)** Can hybrid classifiers combining EEG and pupillometric data improve approaches that solely rely on EEG signals?
- **(RQ4)** What impact does reducing the EEG setup, e.g., for enhanced usability, have on the presented error decoding performance?

## Methods

### Participants

Nineteen volunteers (27.6 ± 2.3 years, mean ± standard deviation (SD), seven female) took part in the study. The participants were free of any known neurological diseases and had normal or corrected-to-normal vision. Thirteen participants self-reported having very little or no experience with HMDs. After instruction, all participants gave written informed consent to take part in the study. The study was approved by the ethical review committee of Graz University of Technology and conducted according to the Declaration of Helsinki (1975). All participants received vouchers worth 20 euros as compensation.

### Experimental procedure

**Figure 1:**
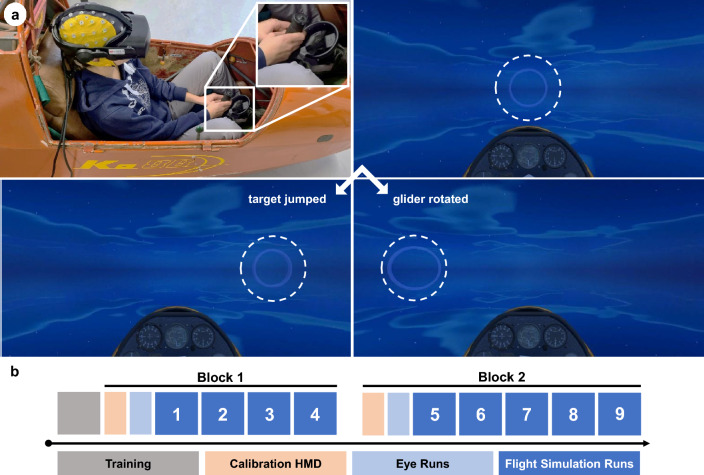
Experimental design. (**a**) Experimental paradigm and setup. Top left: Participant sitting in the immobile glider wearing an EEG cap and the HMD. The HMD controller is attached to the control stick of the physical glider. Top right: The virtual glider is moving toward a target (light blue ring) straight ahead of it. Bottom left: The error event *target* was triggered, the target jumped to its right. Bottom right: The glider rotated to its right in the *passive* condition. Please note that in the *target* condition, only the ring was relocated, however, in the *passive* (and *active*) condition, the participant’s whole field of view changed. The dashed lines were added for illustration purposes only and were not visible to the participants. (**b**) Experimental procedure consisting of two blocks, in which participants completed four (block 1) to five (block 2) flight simulation runs. Data for attenuating ocular artifacts were recorded at the beginning of each block (eye runs). Participants could familiarize themselves with the flight simulation before the recordings.

Participants were comfortably seated in the cockpit of an immobile glider (Ka 8B, Alexander Schleicher GmbH & Co, Germany), as depicted in Fig. 1a. The virtual flight simulation was displayed using an HP Reverb G2 Omnicept HMD (HP, CA, USA), the VR and the paradigm were designed in Unity (https://unity.com/). The experiment was divided into two blocks, each consisting of three phases, i.e., (i) calibration of the eye-tracker of the HMD, (ii) data collection for the eye-artifact removal (eye runs)^38^, and (iii) nine flight simulation runs (four in block 1 and five in block 2, see Fig. 1b). Participants had short breaks of approximately one to five minutes between each flight simulation run and a long break of around ten minutes between the blocks. Participants removed the HMD only once during the long break to minimize the risk of electrode displacements or loss of contact with the scalp. We checked the impedances again before the recordings of block 2. To familiarize themselves with the task, participants completed up to two flight simulation runs before block 1.

To reduce muscle artifacts, participants were instructed to restrict their movements to those necessary for the task, e.g., avoid swallowing or extensive blinking. For that purpose, also the steering interface was designed such that the glider could be steered with minimal movements.

#### Eye runs

EEG recordings are commonly contaminated with artifacts related to eye movements and blinks. We used the sparse generalized eye artifact subspace subtraction algorithm (SGEYESUB)  to reduce these artifacts in the EEG signals. For this purpose, we recorded EEG and gaze data while participants intentionally produced eye-related artifacts, i.e., horizontal and vertical eye movements, and blinks. These signals were used to fit models utilized to attenuate ocular artifacts in the EEG. We refer to the original work for a detailed description of the algorithm^38^. Since their proposed paradigm was designed for a 2D screen, we replicated it in Unity to display it on the HMD. We collected data for the SGEYESUB in two eye runs, one before the first flight simulation run and one after the break. Each eye run took approximately five minutes.

#### Flight simulation runs

Participants could navigate the virtual vehicle using the control stick of the physical glider, to which one HMD controller was attached, as depicted in Fig. 1a. The interaction with the physical glider was meant to increase the realism of the simulation.

The virtual glider moved forward at a constant speed, participants could steer it to the left, right, up, and down. Participants aimed to steer the glider through targets (light blue rings), as shown in Fig. 1a. As participants passed one target, the next one appeared either straight ahead of them, or vertically or horizontally displaced at a fixed angle (6° for vertical and 20° for horizontal displacements, relative to the previous target). In total, one flight simulation run consisted of 70 targets. In 30% of the targets (randomized), one of three possible error events was triggered, such that each of the following error conditions was presented seven times per run (21 error trials per run in total):

- **Target:** In the *target* condition, the target suddenly jumped to either the left or the right, approximately 1.6 s before the glider would have passed it.
- **Passive:** *Passive* interaction errors were triggered 1.1 s to 1.8 s before the glider would have reached the next target. When triggered, the glider suddenly rotated horizontally for 0.6 s, mimicking an unintended turn to the left or right. These errors were only triggered when the next target was straight ahead, not requiring active steering to reach it. Hence, we considered the participants to be in a passive state.
- **Active:** Like the *passive* interaction error, but only triggered before displaced targets, i.e., targets that required active steering to reach them.

The remaining 49 targets per run were considered *correct*, i.e., no error was triggered. The timing of the errors and the positioning of the targets were randomized to obviate adjustments to the simulation. On average, one flight simulation run took approximately 4 min and 45 s, hence a new target appeared roughly every 4 s. A video of the experiment can be found in the supplementary material (Supplementary Video 1).

### Data acquisition

We acquired multimodal physiological data, i.e., EEG, electrocardiography (ECG), and pupillometric data. EEG was recorded with a 63-channel amplifier (eego^TM^sports, ANT Neuro, The Netherlands) at 512 Hz. We positioned the electrodes according to the 10–5 international system at Fp1, Fp2, AF3, AFz, AF4, F7, F5, F3, F1, Fz, F2, F4, F6, F8, FT7, FC5, FC3, FC1, FCz, FC2, FC4, FC6, FT8, FFC3h, FCC1h, FCC2h, FCC4h, T7, C5, C3, C1, Cz, C2, C4, C6, T8, CCP3h, CCP1h, CCP2h, CCP4h, TP7, CP5, CP3, CP1, CP2, CP4, CP6, TP8, P7, P5, P3, P1, Pz, P2, P4, P6, P8, PO3, POz, PO4, O1, Oz, and O2. CPz and AFz were used for reference and ground electrodes, respectively, as in previous works^17,39^. When mounting the EEG electrodes, we made sure that the impedances between the scalp and electrodes were below 10 kΩ. We additionally checked the EEG and ECG signals visually and monitored them throughout the experiment. ECG was recorded with a chest strap (Polar H10, Polar Electro, Austria) at 130 Hz. Gaze direction and pupil size were recorded with the HMD at 120 Hz. We utilized lab streaming layer (https://github.com/sccn/labstreaminglayer) to record and synchronize physiological data and events from the experimental paradigm.

### Data preprocessing

Data were preprocessed and analyzed offline in Matlab R2022a (The MathWorks, MA, USA) incorporating the EEGLAB toolbox (v2022.0)^40^. Statistical tests were run in Python 3.9.12^41,42^.

#### Electroencephalographic data

First, we filtered the EEG between 0.4 and 30 Hz (Butterworth, 4th order, non-causal) and used a notch filter at 50 Hz and 100 Hz to remove power line noise. Next, we applied the SGEYESUB algorithm to correct for blinks and eye movement-related artifacts, as described in the section *Eye runs*. Since various studies concluded that particularly lower frequencies carry information related to error processing^43,44^, we applied another bandpass filter at 1 Hz to 10 Hz (Butterworth, 4th order, non-causal). We resampled the data to 64 Hz to reduce computational effort^16^ and removed the frontopolar and anterior frontal channels to minimize residual contamination from ocular artifacts.

The data from the remaining 58 channels were segmented into trials of 1.5 s (from 0.5 s before to 1 s after error onset). Correct trials were extracted [1.5, 3] s after passing the previous target. At this point, an average of 180 error trials and 422 correct trials were available per participant. Contaminated trials, e.g., due to artifacts stemming from muscular activity or bad channel connectivity, were rejected based on amplitude threshold (exceeding ± 35 µV), kurtosis, and abnormal joint probability^45^. We set the threshold to 5 ⋅ *SD* for the last two. The remaining epochs were visually inspected. We identified bad channels based on both visual inspection and variance. For the last, we computed the first and third quartile (*Q1*, *Q3*) and the interquartile range (*IQR*) of the channel variances and spherically interpolated channels with variances exceeding *Q3* + 1.5 ⋅ *IQR*. On average, we rejected 12 ± 5% of the error trials and 12 ± 3% of the correct trials. We interpolated 1.5 ± 1.6 channels per participant (all are mean ± *SD*).

#### Pupillometric data

As a first step, we removed blinks in the pupil diameter data through linear interpolation. We resampled the data to 128 Hz and applied a bandpass filter between 0.1 and 10 Hz (Butterworth, 4th order, non-causal). When analyzing the data recorded in the eye runs, we found a dependency of the pupil size on the horizontal gaze angle. This dependency is most likely a result of the so-called pupil foreshortening error (PFE). PFE is mainly influenced by the apparent change in the shape of the pupil when moving away from a camera, which is a well-known problem in the measurement of pupil sizes with HMDs^47^. To correct this error, we used all segments of the eye runs^38^ in which participants horizontally moved their eyes following a visual stimulus. We fitted a 3rd order regression function to estimate the dependency of the pupil size on the gaze direction, separately for each participant and both eyes. This dependency was considered a consequence of the PFE and subsequently subtracted from the pupil size data recorded in the flight simulation runs. After correction, we averaged the signals from both eyes and cut the resulting signal into trials of 1.5 s, as described in the previous section. We removed noisy trials based on variance. Therefore, we calculated *Q1*, *Q3*, and *IQR* of the trials’ variances and removed trials with a variance greater than *Q3* + 1.5 ⋅ *IQR*. Trial rejection was performed separately for erroneous and *correct* trials, we rejected on average 6 ± 2% of the trials for each (mean ± *SD*). Finally, we corrected each trial by subtracting the mean of the baseline, i.e., [− 0.5, 0] s before the error onset, or with the mean of the entire correct trial, respectively.

#### Electrocardiographic data

The RR interval was computed as the time difference between the R peaks of every pair of two consecutive QRS complexes, the corresponding HR for each peak is its inverse^48^. We linearly interpolated the HR values between two complexes and segmented the data into epochs of 3.5 s, i.e., [− 0.5, 3] s relative to the error onset. Subsequently, we corrected each trial by subtracting its respective mean from the baseline window, i.e., [− 0.5, 0] s. Finally, we averaged the HR changes for each participant and error type to obtain the grand average HR changes.

### Asynchronous multiclass error decoding approaches

In the following section, we describe in detail our error decoding strategies based on the preprocessed physiological data, i.e., EEG and pupil size. We investigated two different data fusion techniques to combine the information from both modalities, which will be referred to as simple fusion (SF) and Bayesian fusion (BF). However, we did not consider ECG data in these approaches. The high trial-to-trial variability, resulting from respiratory sinus arrhythmia^49^, made error decoding based on HR variations infeasible (see *Limitations*).

#### EEG-based error decoding

Before classification, we resampled the data to 32 Hz. In our multiclass error decoding approach, we used fairly balanced class sizes by keeping all preprocessed error trials and randomly choosing the number of *correct* trials equal to the average number of trials in the error classes (four classes in total)^7,24^. We used a 10 times 5-fold cross-validation (CV) to divide the data of each participant into a training and a test set. Error decoding was performed using a shrinkage linear discriminant analysis (sLDA) classifier^50^, which is a commonly used method in classification problems with ERPs^51^. To train the classifier, we slid a window of variable length through the trials. For each window, we trained the classifier on the training set and evaluated it on the test set, generating an output every 31.25 ms. Window lengths included 1 sample, 125 ms (4 samples), 250 ms (8 samples), and 500 ms (16 samples). As features, we used the amplitude values of the trials of the training set within the current window. Hence, we extracted *C* times* W* features for classification, where *C* is the number of EEG channels (1, 3, or 58, see below) and *W* is the window length (1, 4, 8, or 16). Subsequently, we performed principal component analysis (PCA) to reduce the number of features and kept those that explained 99% of the variance. For each participant, we averaged the classification accuracies from the 50 folds to calculate the 19 participants' mean accuracies.

In addition to the variation of window sizes, we analyzed the performance of reduced electrode setups on the decoding accuracy. We pre-selected the channels for these setups based on extensive literature that reports them to be of particular relevance for error decoding^22^. Subsequently, we decided to test three different layouts:

- **1-channel layout:** FCz,
- **3-channel layout:** FCz, Cz, Pz,
- **Full layout:** all 58 electrodes.

#### Error decoding based on pupil size

For this approach, we performed the same analysis as described above for the EEG data using the preprocessed pupil size signals. In addition to this personalized classification approach, we investigated if classifiers trained with pupil data from one participant can be transferred to data from another participant, i.e., generic classification. Here, the training set consisted of the balanced data from all but one participant (leave-one-out), and the data from the remaining participant was the test set. The remainder is like in the personalized classification approach.

#### Simple fusion

The SF approach is mostly analogous to the classification based on EEG signals. However, for SF we treated the pupil size as an additional data channel, without making any distinction to other EEG channels. Before performing PCA, we made sure that both EEG and pupillometric data had zero mean and equal variance.

#### Bayesian fusio*n*

A confusion matrix ${C}_{k}={\left({n}_{ij}^{\left(k\right)}\right)}_{i,j=1}^{M}$ comprehensively characterizes the reliability of a classifier. Each row corresponds to one of the *M* true classes *c*_*1*_, …, *c*_*M*_ the sample was drawn from, and each column corresponds to the class predicted from the classifier *k*. Hence, *n*_*ij*_^*(k)*^ is the number of samples from class *i* that classifier *k* assigned to class *j*. Given the class prediction *e*_*k*_ from each of the *K* classifiers, the Bayesian belief measure^52,53^ is defined as

1 $$O\left( {c_{i} } \right) \propto P\left( {c_{i} } \right)\mathop \prod \limits_{k = 1}^{K} P\left( {c_{i} | e_{k} = c_{j} } \right),$$

with

2 $$P\left( {c_{i} | e_{k} = c_{j} } \right) = \frac{{n_{ij}^{\left( k \right)} }}{{\mathop \sum \nolimits_{i = 1}^{M} n_{ij}^{\left( k \right)} }},i = 1, \ldots ,M;j = 1, \ldots ,M,$$

where *P(c*_*i*_*)* is the prior probability of the *i-th* class and *P(c*_*i*_* | e*_*k*_ = *c*_*j*_*)* is the probability that the true class is *i* when the classifier *k* predicts class* j*. The class that maximizes *O* is the output of the fused classification.

In our case, we implemented *K* = 2 classifiers, i.e., classification based on EEG and pupillometric data, which predicted *M* = 4 classes (*target*, *passive*, *active*, and *correct*). The computation of the outputs of the individual classifier follows the descriptions in the sections *EEG-based error decoding* and *Error decoding based on pupil size*. The confusion matrices *C*_*k*_ which were used to obtain the conditional probabilities *P(c*_*i*_* | e*_*k*_ = *c*_*j*_*)* were computed from the training set in the 10 times 5-fold CV and evaluated on the corresponding test set.

### Statistical analysis

To assess which classifiers performed better than chance^54^, we used a cumulative binomial distribution. Given the number of trials *n* and the number of conditions *c* = 4, the probability of randomly predicting the condition correctly *k* times is calculated as follows^55,56^:

3 $$P\left(k\right)={\sum }_{i=k}^{n}\left(\genfrac{}{}{0pt}{}{n}{i}\right)\cdot {\left(\frac{1}{c}\right)}^{i}\cdot {\left(\frac{c-1}{c}\right)}^{n-1}.$$

We present the significance threshold (*α* = 0.05) computed as the average of the subject thresholds (Figs. 4, 5, 7).

We performed Wilocoxon signed-rank tests based on participant-level data to compare the peak error-related pupil dilations [0.5, 0.8] s after the error events with the maximum values in correct trials within the same window. Similarly, we compared the peak error-related HR decelerations within the window [0.7, 1.7] s with the minimum values in* correct* trials. We used the false discovery rate (FDR) procedure to correct for multiple testing (*α* = 0.05). Windows are based on the grand average responses in Fig. 3.

Further, we used Wilcoxon signed-rank tests to compare the participants’ peak accuracies (personalized vs. generic) for each window length based on the pupil size (Fig. 4). Again, we performed FDR correction (*α* = 0.05).

Similarly, we compared results from EEG and hybrid approaches by performing sample-wise Wilcoxon signed rank tests with subsequent FDR correction (*α* = 0.05) for each layout and window length (Fig. 5). Finally, to compare the peak accuracies and mean correctly classified samples using the approaches SF, BF, and EEG only, we performed a Friedman test for each window length (Fig. 6). We corrected the *p*-values using the FDR procedure. Subsequently, we performed Nemenyi tests for post-hoc comparisons where the omnibus tests revealed statistically significant differences (*p* < 0.05).

## Results

### Physiological correlates of error processing

**Figure 2:**
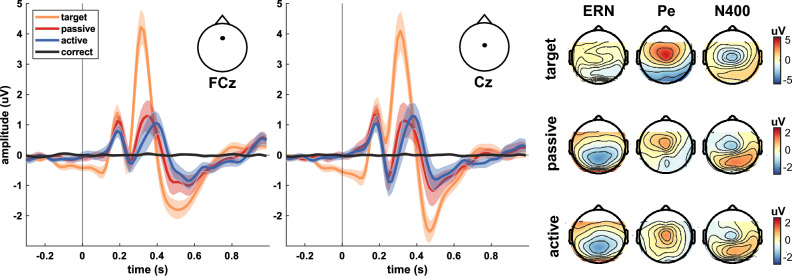
Grand average neurophysiological results. Shown are the ErrPs at FCz and Cz for the classes *target* (yellow), *passive* (red), *active* (blue), and *correct* (black) after the error onset at *t* = 0 s. Shaded areas show the SEM. Topographical distributions of the ErrPs for *target* (top row), *passive* (middle row), and *active* (bottom row) are given at the ERN (*t* = 235–265 ms), Pe (315–390 ms), and N400 (470–500 ms).

To investigate **RQ1**, we analyzed the physiological response to the error conditions in detail. In Fig. 2, we present the grand average EEG results from 19 subjects for FCz and Cz (mean ± standard error of the mean (SEM)) for the error conditions *target*, *passive*, and *active*, and the *correct* condition after re-referencing to the common average^46^.

For the ErrPs at FCz, we found an initial positive peak 188 ms after error onset in all error conditions. Average amplitudes of P1 are 1.33 µV for *target*, 1.03 µV for *passive,* and 0.748 µV for *active*. *Target* errors (yellow) elicited a subsequent ERN at 234 ms (− 0.401 µV) and Pe at 313 ms (4.16 µV). Components of the ErrPs elicited by *active* errors (blue) are delayed compared to the *passive* ones (red). In the *passive* condition, we found an ERN at 250 ms (− 0.274 µV) and a Pe at 344 ms (1.30 µV). *Active* errors elicited an ERN at 266 ms (− 0.331 µV) and a Pe peaking at 391 ms (1.08 µV). We additionally found an N400 in all conditions peaking at 469 ms (*target*), 484 (*passive*), and 500 ms (*active*). A negative deflection before the error onset appears in the *target* condition, *correct* trials (black) do not show any distinct error-related response. Additionally, we show the topographical distribution at the ERN, Pe, and N400 revealing frontal and parietal activity.

**Figure 3:**
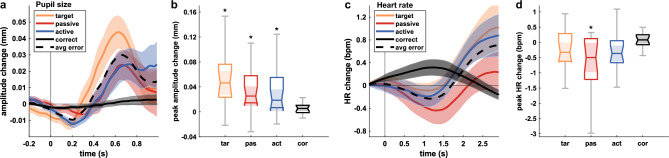
Grand average pupillometric (left) and cardiac (right) responses. (**a, c**) Physiological responses relative to the baseline period for the classes *target* (yellow), *passive* (red), *active* (blue), and *correct* (black). The dashed lines show average error-related changes. Shaded areas indicate the SEM. Error onset is a* t* = 0 s. (**b, d**) Distributions for the peak responses per participant within the windows [0.5, 0.8] s (pupil size) and and [0.7, 1.7] s (HR). Significant differences between each error condition and *correct* are indicated (*p < 0.01).

Figure 3 shows the grand average pupillometric and evoked cardiac responses. Pupil dilations peak later than ErrPs, i.e., after 625 ms for *target*, after 656 ms for *passive,* and after 734 ms for *active*. Pupil size changes are relative to the baseline period, as described in 3.4.2. All error-related cardiac responses show an initial deceleration of the HR (relative to the baseline), followed by an acceleration 1 to 1.5 s after the error onset. The average error-related changes are shown in black (dashed line). Peak error-related pupil dilations differ significantly from *correct* for all error conditions (*target*: *p* = 0.002, *passive*: *p* = 0.005, *active*: *p* = 0.006). HR decelerations are significant after *passive* errors (*p* = 0.008). Further analyses of the error-related physiological responses can be found in our previous works^57,58^.

### Multiclass classification

**Figure 4:**
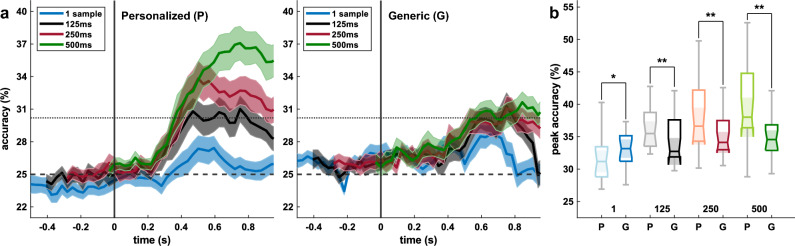
Grand average classification results based on the pupil size only. Error onset is at *t* = 0 s. (**a**) Classification results using personalized classifiers (left) and generic classifiers (right) for four window lengths, i.e., 1 sample (blue), 125 ms (black), 250 ms (red), and 500 ms (green). Shaded areas indicate the SEM. Chance level (25%, dashed line) and significance threshold (30.2%, dotted line) are given. (**b**) Distributions of the peak accuracies of the 19 participants’ results. Significant differences between personalized (P, light colors) and generic (G, dark colors) classification are indicated (*p < 0.05, **p < 0.01).

Regarding **RQ2**, the classification results using only pupillometric data are illustrated in Fig. 4. In the personalized classification approach (P), error decoding with a window length of 250 ms and 500 ms yielded accuracies above the significance threshold (dotted lines), with peak accuracies of 33.8% at *t* = 500 ms and 37.1% at *t* = 750 ms, respectively. None of the generic classifiers (G) could perform better than random (Fig. 4a). The dashed lines show the theoretical chance level of 25%, the dotted lines show the average significance threshold of 30.2%.

We found the peak accuracies of the personalized classifiers to be significantly better than the generic results for the windows 125 ms (*p* = 0.007), 250 ms (*p* = 0.003), and 500 ms (*p* = 0.003). Peak accuracies also differ in the single-sample approach (*p* = 0.030). Distributions of the peak accuracies are shown in Fig. 4b.

**Figure 5:**
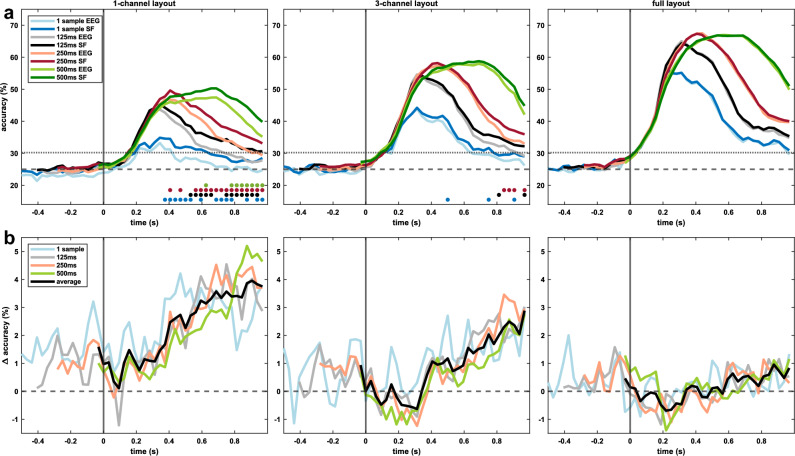
Comparison of the grand average classification results of EEG only and SF (simple fusion). Error onset is a *t* = 0 s. (**a**) Grand average multiclass classification results for three layouts, i.e., 1-channel, 3-channel, and full layout, and four window lengths, i.e., 1 sample (blue), 125 ms (black), 250 ms (red), and 500 ms (green). Classification results of the approach using only EEG data are depicted in brighter colors, SF in darker colors. Chance level (25%, dashed line) and significance threshold (30.2%, dotted line) are given. Dots indicate statistically significant samples (*p* < 0.05) for the respective comparisons, i.e., EEG only vs. SF. (**b**) Difference between the accuracies of SF and EEG. Differences between SF and EEG for each window size are shown in bright colors, the mean differences for each layout are shown in black. For instance, the green lines show the improvements of the hybrid approach using a 500 ms window.

Since we could not find significant results in the generic classification approach, we focus on personalized error decoding in the remainder of this work. To answer **RQ3**, we compare the classification results based on EEG only with the SF approach in Fig. 5. Figure 5a shows the classification results for all considered layouts and window lengths (1 sample: blue, 125 ms: black, 250 ms: red, 500 ms: green). Results from the hybrid approach are presented in darker colors, and accuracies obtained from EEG only are brighter. Significant improvements (*p* < 0.05) in the accuracies of the hybrid decoders are mainly found in the 1-channel layout, starting approximately 500 ms after error onset. In Fig. 5b, we highlight the influence of the data fusion by presenting the accuracy gains as the difference between SF and EEG, showing average improvements of up to 4% in the 1-channel layout and about 2.5% in the 3-channel layout. In the full layout, no improvements could be found.

**Figure 6:**
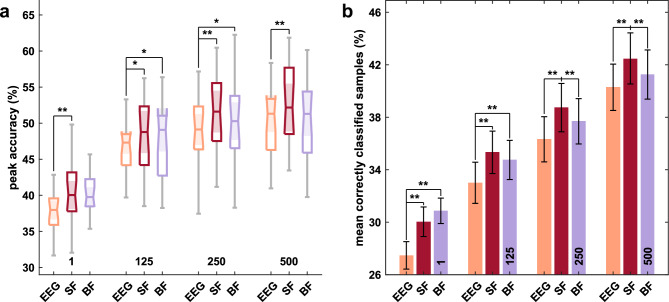
Classification results for the 1-channel layout. Average peak accuracies (**a**) and correctly classified samples (**b**) are presented for the decoding methods EEG only (orange), SF (simple fusion, red), and BF (Bayesian fusion, purple). Window lengths are given at the bottom part of each subfigure. Significant differences are indicated (*p < 0.05, **p < 0.01).

As the 1-channel layout is the setup with the best usability and yielded the greatest improvements in the hybrid error decoding approach, we analyzed this setup in more detail for **RQ4**. Figure 6 shows the classification results for EEG only and compares them to both hybrid approaches, i.e., the simple (SF) and Bayesian fusion (BF). In Fig. 6, we investigate the performance regarding two metrics of interest, i.e., the peak accuracies (Fig. 6a) and the correctly classified samples after error onset^59^, i.e., in the segment [0, 1] s (Fig. 6b).

For the peak accuracies, Friedman test revealed significantly different results for the 1 sample approach (χ^2^(2) = 10.2, *p* = 0.010). Post-hoc test showed that SF achieved better accuracies than EEG (*p* = 0.006). Using the 125 ms window (χ^2^(2) = 7.89, *p* = 0.019), post-hoc tests found SF and BF to perform better than EEG (both *p* = 0.04). Significant differences were also found for the 250 ms window (χ^2^(2) = 9.78, *p* = 0.010), particularly for EEG vs. SF (*p* = 0.010) and SF vs. BF (*p* = 0.040), and for the 500 ms window (χ^2^(2) = 9.58, *p* = 0.010), SF outperformed EEG (*p* = 0.006).

For the correctly classified samples, Friedman test found differences for the 1 sample approach (χ^2^(2) = 30.6, *p* < 0.001), both fusion approaches yielded better results than EEG (*p* = 0.001). We further found differences for the 125 ms window (χ^2^(2) = 27.3, *p* < 0.001), in particular, SF (*p* = 0.001) and BF (*p* = 0.006) performed better than EEG. Further, for the 250 ms window, we found results to differ significantly (χ^2^(2) = 27.3 *p* < 0.001) between SF and EEG (*p* = 0.001) and SF vs. BF (*p* = 0.006). For the 500 ms window, Friedman test revealed differences (χ^2^(2) = 22.8, *p* < 0.001) too. Here, again SF performed better than EEG (*p* = 0.001) and BF (*p* = 0.006).

**Figure 7:**
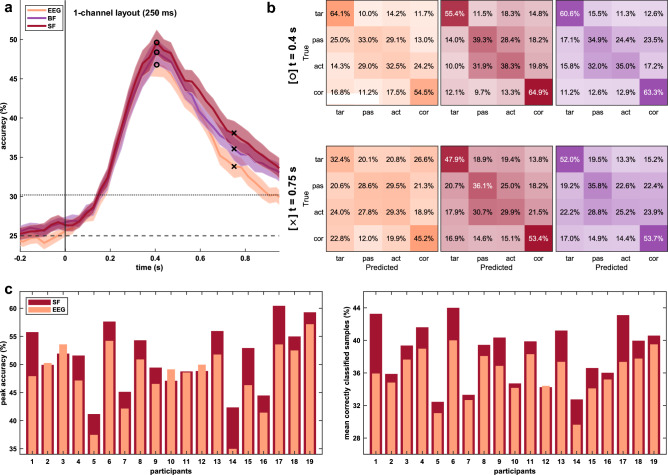
Classification results for the 1-channel layout and the 250 ms window. (**a**) Grand average classification results for the approach using only EEG data (orange), SF (simple fusion, red), and BF (Bayesian fusion, purple). Shaded areas indicate the SEM. Chance level (25%, dashed line) and significance threshold (30.2%, dotted line) are given. (**b**) Row-normalized confusion matrices for the time points indicated in (**a**), i.e., 400 ms (O, top row) and 750 ms (✕, bottom row) after the error onset at *t* = 0 s. (**c**) Results per participant for peak accuracy (left) and mean correctly classified samples (right) computed for EEG only (orange) and SF (red).

As an example, we further analyzed the decoder performance using the 250 ms window in the same setup in Fig. 7 in more detail. Figure 7a compares the accuracies of the three classifiers (EEG, SF, BF). BF and SF (peak accuracy of 48.4% and 49.6%, respectively) outperformed EEG only (46.8%). Figure 7b shows the confusion matrices at two points of interest. We chose the time point of the peak accuracies *t* = 400 ms (marked with O) and *t* = 750 ms (marked with ✕) after error onset. The sliding windows at time points include the most prominent components of the neural and pupillometric responses, i.e., the Pe and the maximum pupil dilations. Here, it is particularly visible that the accuracies for EEG only decline faster than for the fusion approaches, emphasizing the delayed impact of the pupil size signals compared to the ErrPs. For example, at *t* = 750 ms less than a third of the *target* trials were classified correctly using EEG data only (32.4%), whereas in SF and BF around half of these trials were assigned correctly (47.9% and 52.0%). Figure 7c illustrates the classification results at the participant-level. After adding pupillometric information, both peak accuracies (left) and mean correctly classified samples (right) increased between 3 and 8% in ten and six participants, respectively.

## Discussion

### VR flight simulation

The goal of our experimental setup was to create a realistic experience for the participants. For that, we seated the participants in a physical glider, which was also the template for the glider they navigated in the simulation. Further, we provided a realistic steering interface, i.e., the control stick of the physical glider, to which we attached the control stick of the HMD. We emphasize the design choices of the experimental setup, as Slater and Wilbur^60^ describe immersion as the technical affordances that create the illusion of being part of the VE. Efforts made to create an immersive experience aim to cause the feeling of presence, i.e., the user’s feeling of *being in* the VR. Users with a strong sense of presence feel more engaged in the interaction with the virtual world^61^. Engagement is known to modulate error-related activity. Hajcak et al.^62^ motivated participants with monetary rewards for correct responses and found the ERN to be significantly larger after errors committed in high-value stimuli. The relevance of motivational significance has also been reported for error-related pupil responses^63,64^. However, we did not assess the participant’s engagement to validate how successful our efforts to create a realistic environment were.

A crucial point for assessing error-related pupil dilations is maintaining constant luminosity. Hence, we carefully designed the VE such that no sudden changes in luminosity occur. We only allowed marginal variations in small sections of the scenery that are necessary to facilitate the sense of realism (e.g., positioning of background clouds, as visible in Fig. 1 and in Supplementary Video 1). However, we randomized not only the direction of the errors (e.g., glider rotation to the left or right) but also their order and timing. Thus, each run for each participant was different to avoid systematic dependencies between pupil response and VE. For example, target jumps, which were triggered without any change of the background scenery, elicited the strongest pupil dilations.

### Error-related physiological responses

Grand average neurophysiological results show ErrPs with components commonly reported in erroneous HCIs^6^ (**RQ1**). Interestingly, ErrPs caused by *active* errors are delayed compared to correlates of *passive* errors. Grand average error positivities of *passive* and *active* errors differ by almost 50 ms, resulting from the participants’ activity. Comparable findings have been reported by Lopes-Dias et al.^65^, who found a delayed response after errors with a masked error onset. Additionally, we found a negative deflection in the *target* condition, starting slightly before the error onset. Brunia and Damen^66^ demonstrated that stimulus anticipation is reflected by a slow negative deflection, i.e., the stimulus-preceding negativity. Subsequently, participants anticipated the jumps of the targets because of the missing randomization of the error onset. However, unintended glider rotations, as in the *passive* and *active* conditions, were triggered in a randomized manner, hence, no anticipation-related effects are visible.

Error-related pupil dilations are in alignment with previous findings regarding their latency, peaking approximately 600 ms after error onset^31^, and their sensitivity to the evaluation of different types of errors^33^. Interestingly, we could identify a delay in the error-related pupillometric responses during active task executions, similar to the findings in the EEG correlates. Post-error pupil dilations have been described as a consequence of the orientation response (OR)^67^. The OR is an immediate reaction of an organism to unexpected changes in its environment, which causes activations of central and autonomous physiological systems^30,32,35^. Danev and de Winter^34^ demonstrated a deceleration of the HR after erroneous responses and suggested this to be a manifestation of the OR. Later works confirmed their findings^32,35^, which are consistent with our results. The following HR accelerations after approximately 1.5 s (Fig. 3) are likely influenced by other factors, such as suddenly appearing targets of the next trial, and hence do not necessarily reflect error processing. However, similar findings were also reported earlier^32^.

### Multimodal error decoding

A primary goal of this work was to analyze the possibility of error decoding solely using pupil responses (**RQ2**), as we are not aware of any prior attempts. Above-chance multiclass classification was possible with the two longest windows, i.e., 250 ms and 500 ms, peaking at 34% and 37%, respectively. Moreover, we tested if a participant-to-participant transfer of such classifiers is possible. This would be of particular interest, since generic classifiers eliminate a major drawback of personalized approaches, i.e., the usually very time-consuming calibration phase of the classifiers^11,68^. However, our generic classification results based on pupillometric data did not exceed the significance threshold, which indicates a large inter-subject variation in the pupillometric responses. This difficulty has already been mentioned earlier^69^.

Based on these findings, we studied the impact of the fusion of EEG and pupillometric data (**RQ3**) using personalized classifiers. We investigated the influence of different window lengths, as they are known to impact decoder performances^70,71^. Shorter windows, e.g., using only a single sample, facilitate a higher temporal resolution enabling us to study the distribution of information^72^. Such decoders are more responsive to changes in the underlying data and their performances peak faster. However, classification using longer windows is expected to offer better accuracy, particularly because neural and pupillometric responses can be covered simultaneously. To analyze these advantages and drawbacks, we varied the window lengths from 1 sample to 500 ms, and generally observed the hypothesized impacts. Considering, e.g., the 1-channel layout, the peak accuracy was reached after 340 ms using the 1 sample window, and after 410 ms using the 250 ms layout, at the cost of poorer performance (about 35% and 50%, respectively). Using 500 ms yielded a similar performance (50%) at a slower response time (660 ms). Increasing the number of channels leads to better performances, i.e., peak accuracies of 59% (3-channel) and 67% (full layout).

A fair comparison between error decoding performances reported in different works is difficult since considerable differences in data processing, classification methodology, and evaluation metrics might exist. Still, commonly reported accuracies lie between 70 and 80% for binary classification tasks, i.e., error vs. correct^22^. Our presented peak accuracies of up to 67% obtained with the full layout are marginally below that. However, this performance was achieved in a multi-class problem, which has rarely been demonstrated. Nevertheless, the presented classification accuracies are insufficient for practical applications. Hence, our results suggest that a transfer to real-world scenarios would likely involve a reduction to, e.g., two classes, to elevate the BCI’s performance.

Error decoding on a minimal subset of channels (**RQ4**) has been attempted in previous studies, e.g., using only one electrode from a portable EEG headset^37^, which is a necessity to make a system usable outside of the lab. Reducing the number of channels led to an expected drop in accuracy. We show that this performance drop can be partially abated by adding pupil size data, which can be measured using many HMDs with practically no additional effort. In both reduced electrode setups, we found significant improvements using hybrid approaches compared to EEG only, mainly starting 500 ms after error onset, which can be explained by the latency of the pupillometric responses. Peak improvements were up to 4% for the 1-channel layout and approximately 2.5% for the 3-channel layout. Consequently, the results of this work suggest that a reduced setup incorporating pupil data could be a trade-off for potential end users.

The second implemented hybrid approach (BF) is based on Bayesian probabilities and has already been tested in fusing multimodal physiological data, e.g., EEG and muscular activity^59,73^. Surprisingly, the simple data fusion approaches performed better than BF in most cases. This might be explained by an insufficiently small test set used to compute the confusion matrices, causing possibly weak estimators for the conditional probabilities. However, Leeb et al.^59^ reported similar results for a simple approach and Bayesian fusion too.

### Limitations

The presented offline approach for asynchronous classification is not directly transferable to an online scenario with continuous decoder evaluation. Offline analysis of physiological data allowed us to utilize non-causal filters, which compensate for group delays. Non-causal filters are not applicable in online experiments, however, the choice of filter might influence the resulting ErrPs considerably^4,16^. Nonetheless, we wanted to suggest tools that allow for online correction of eye-related artifacts, which are inevitable in real-world settings, such as the presented game-like flight simulation. For this purpose, we applied the SGEYESUB, which can be used for offline and online correction of contaminated data^38^. Moreover, continuos error decoding comes with additional challenges, including misinterpreting other stimuli or artifacts, e.g., stemming from electromyographic activity, as errors. Such challenges have to be carefully considered in the design and training of classifiers. Previous works implemented them successfully^4^.

Further, our classification approaches solely rely on features from the temporal domain. We did not examine if including additional features, such as features obtained from the frequency domain, has the potential to increase classification accuracy, as proposed earlier^16,74^. However, multiple works have reported that a combination of temporal and frequency domain features did not yield improved performance^4,7^. Interestingly, time–frequency domain features have been demonstrated to be suitable for error decoding^75^ and possibly deserve more attention in future studies. We decided to analyze EEG signals in the low frequencies only, i.e., 1 Hz to 10 Hz, as error-induced low-frequency activity has been commonly reported^43,44^. Völker et al.^76^ demonstrated that error processing modulates activity in the gamma band, which could also be considered in error decoding. Similarly, we did not investigate which pupillometric features, in addition to temporal features, could contribute to error decoding. As, to the best of our knowledge, there is no previous research on error decoding on pupillometric data, investigations on this should be subject to future works.

Finally, we did not include error-related HR changes in our hybrid classification approach since influences of the breathing cycle^49^ made error decoding with them infeasible. However, there exist works that successfully removed respiratory influences from the HRV. Commonly, such approaches require the acquisition of additional physiological signals, e.g., respiration signals, as reference^77^. Papers that implemented methods that solely rely on ECG data are scarce^78^. Future works should consider these deliberations in their experimental designs.

## Conclusion

In this work, we studied the correlates of error processing in multimodal physiological signals, i.e., EEG, ECG, and pupil size. We decoded three different types of errors utilizing EEG signals and focused on studying BCIs in setups with improved practical usability, i.e., with a minimal number of electrodes. Further, we assessed the potential impact of additional pupil size information in a hybrid classification approach and found significant improvements compared to error decoding with one EEG channel. Such minimal setups are of interest in the context of HCIs, particularly using HMDs, since the latest devices offer straightforward access to multimodal physiological data. Continuous online error decoding using these signals, potentially including real-time adaptations of the VE informed by the error decoder, should be aimed in future studies.

### Supplementary Information

Supplementary Video 1.

## Data Availability

The data provided in this study are available upon reasonable request from the corresponding author.
